# Acute Kidney Injury Predictors and Outcomes after Cardiac Surgery in Children with Congenital Heart Disease: An Observational Cohort Study

**DOI:** 10.3390/diagnostics12102397

**Published:** 2022-10-02

**Authors:** Georgios Kourelis, Meletios Kanakis, George Samanidis, Kimon Tzannis, Dimitrios Bobos, Theofili Kousi, Sotiria Apostolopoulou, Felicia Kakava, Konstantinos Kyriakoulis, Stavroula Bounta, Spyridon Rammos, John Papagiannis, Nickolas Giannopoulos, Stylianos E. Orfanos, George Dimopoulos

**Affiliations:** 1 Pediatric Cardiac and Adult Congenital Heart Disease Intensive Care Unit, Onassis Cardiac Surgery Center, Andrea Syggrou 356 Av., 17674 Kallithea, Greece; 2 Paediatric Cardiac and Adult Congenital Heart Disease Surgical Department, Onassis Cardiac Surgery Center, Andrea Syggrou 356 Av., 17674 Kallithea, Greece; 3 Department of Adult Cardiac Surgery, Onassis Cardiac Surgery Center, 17674 Athens, Greece; 4 4th Department of Internal Medicine, National and Kapodistrian University of Athens Medical School, 1 Rimini Street, 12462 Athens, Greece; 5 Department of Anesthesiology, Onassis Cardiac Surgery Center, Andrea Syggrou 356 Av., 17674 Kallithea, Greece; 6 Department of Pediatric Cardiology and Adult Congenital Heart Disease, Onassis Cardiac Surgery Center, Andrea Syggrou 356 Av., 17674 Kallithea, Greece; 7 1st Department of Critical Care, National and Kapodistrian University of Athens Medical School, 12462 Athens, Greece; 8 3rd Department of Critical Care, “EVGENIDIO” Hospital, National and Kapodistrian University of Athens (NKUA), 12462 Athens, Greece

**Keywords:** congenital heart disease, acute kidney injury, cardiac surgery, risk factors, children, cardiac intensive care unit

## Abstract

Acute Kidney Injury (AKI) commonly complicates cardiac surgery in children with congenital heart disease (CHD). In this study we assessed incidence, risk factors, and outcomes of postoperative AKI, while testing the hypothesis that, depending on the underlying diagnosis, there would be significant differences in AKI incidence among different diagnostic groups. We conducted an observational cohort study of children with CHD undergoing cardiac surgery in a single tertiary center between January 2019 and August 2021 (*n* = 362). Kidney Disease Improving Global Outcome (KDIGO) criteria were used to determine the incidence of postoperative AKI. Diagnosis was incorporated into multivariate models using an anatomic-based CHD classification system. Overall survival was estimated using Kaplan–Meier curves. Log-rank test and adjusted Cox proportional hazard modelling were used to test for differences in survival distributions and determine AKI effect on survival function, respectively. AKI occurred in 70 (19.3%), with 21.4% in-hospital mortality for AKI group. Younger age, lower weight, longer cardiopulmonary bypass time, preoperative mechanical ventilation and diagnostic category were associated with postoperative AKI. Resolution rate was 92.7% prior to hospital discharge for survivors. AKI was associated with longer duration of mechanical ventilation, ICU and hospital length of stay. AKI patients had significantly higher probability of all-cause mortality postoperatively when compared to the non-AKI group (log-rank test, *p* < 0.001). Adjusted hazard ratio for AKI versus non-AKI group was 11.08 (95% CI 2.45–50.01; *p* = 0.002). Diagnostic category was associated with cardiac surgery-related AKI in children with CHD, a finding supporting the development of lesion specific models for risk stratification. Postoperative AKI had detrimental impact on clinical outcomes and was associated with decreased survival to hospital discharge.

## 1. Introduction

Acute kidney injury (AKI), defined as an abrupt decline in renal function, commonly complicates pediatric cardiac surgery (CS) for congenital heart disease (CHD) while being associated with significant morbidity and mortality [1,2,3,4]. Alarming evidence shows that children developing postoperative AKI have elevated renal biomarkers several years after initial insult, possibly indicating an increased risk for chronic kidney disease [5,6,7,8].

Current evidence suggests multiple factors to be associated with CS-related AKI including younger age, lower weight, cyanotic or univentricular heart defects, higher complexity surgical procedures, use and longer duration of cardiopulmonary bypass (CPB), sepsis, use of nephrotoxic agents, intraoperative hypotension, and postoperative low cardiac output state [1,3]. Few studies have been conducted in children with lesion-specific CHD [9,10,11]. The majority refers to heterogenous populations regarding phenotype and underlying anatomy, rendering the application of their findings to specific lesions questionable [12].

Criteria used to define AKI have undergone several modifications over time. Kidney Disease Improving Global Outcomes (KDIGO) classification system, which integrates preceding RIFLE (Risk, Injury, Failure, Loss of kidney function, End-stage kidney disease) and AKIN (Acute Kidney Injury Network) systems, has been previously used to determine the incidence of postoperative AKI in children with CHD and is probably the most inclusive [2,13,14].

The aim of this observational study was to assess the incidence of postoperative AKI in children undergoing CS for CHD using KDIGO criteria and to investigate possible risk factors associated with it. Additionally, we incorporated diagnosis in our models by utilising an anatomic-based classification system for CHD seeking for possible associations between specific diagnostic groups and AKI. Furthermore, we looked to describe AKI’s association with duration of mechanical ventilation (DMV) and length of stay (LOS), both Pediatric Intensive Care Unit (PICU) and hospital. Finally, we investigated AKI’s impact on survival function.

## 2. Materials and Methods

### 2.1. Study Design

We analysed prospectively collected data from consecutive children undergoing surgery for CHD between January 2019 and August 2021. Eligible patients were patients <18 years old with CHD undergoing surgical correction or palliation, elective or not. Exclusion criteria were unavailable preoperative creatinine levels (*n* = 2), preoperative renal replacement therapy (*n* = 3), and death within 24 h after surgery (*n* = 2).

### 2.2. Data Collection—Definition of Variables

The following data were recorded: gender, surgical age, surgical weight, previous surgery for CHD, preoperative invasive mechanical ventilation, cardiac computed tomography (CT) scan within 7 days preoperatively, balloon atrial septostomy (BAS) preoperatively, need for and CPB duration, need for and aortic cross clamp duration. PICU (total and postoperative) and hospital LOS, DMV (total and postoperative), and in-hospital mortality were recorded as outcome variables. DMV was calculated as [(*extubation date − intubation date*) *+* 1] and LOS as [(*discharge date − admission date*) *+* 1].

Surgical age and weight were presented as continuous and dichotomous variables. Risk Adjustment for Congenital Heart Surgery (RACHS-1) system was used to classify each procedure performed [15]. For multivariate logistic regression, the cohort was divided into 2 groups (RACHS-1 categories 1–2 and RACHS-1 categories 3–6, respectively).

AKI was defined based on KDIGO clinical practice guidelines [13]. A minimum serum creatinine (SCr) of >0.5 mg/dL was used for AKI diagnosis to avoid bias towards classifying AKI as present in young infants [16]. For patients with postoperative AKI, resolution was defined as a drop in SCr to <150% of the preoperative reference value at hospital discharge. A minimum value of >0.5 mg/dL at hospital discharge was additionally required to define non-resolution.

We classified patients into the following diagnostic categories: 1. CHD with shunt between systemic and pulmonary circulation, 2. left heart CHD, 3. right heart CHD, 4. CHD with anomalous origin of great arteries, 5. univentricular lesions, and 6. miscellaneous CHD [17]. Patients with complex CHD were classified based on the lesion demanding earliest surgical intervention. Patients with unclassifiable CHD (*n* = 6) were excluded from multivariate models. Patients classified into categories 4–6 were further grouped together for multivariate models, due to small patients’ number compared with categories 1–3.

### 2.3. Intraoperative Procedure

Anesthesia induction was achieved with either sevoflurane 7–8% for children <8 years old or propofol (2–3 mg/kg) for older children combined with fentanyl (2–3 μg/kg). Muscle relaxation was facilitated with rocuronium (1 mg/kg). All patients were intubated. Anesthesia maintenance was achieved combining inhaled sevoflurane 1–3%, fentanyl boluses and cis-atracurium infusion (0.1 mg/kg/h). All patients received Piperacillin/Tazobactam and Teicoplanin perioperatively. Tranexamic acid (10–30 mg/kg) was given to all patients unless contraindicated.

Three surgeons experienced in pediatric CHD surgery performed all operations. Cases concerning patent ductus arteriosus (PDA), discrete coarctation of the aorta (CoA), and modified Blalock–Taussig shunts were performed without CPB. The rest of the cases were managed using either normothermic or mild–moderate hypothermic CPB. For Hypoplastic Left Heart Syndrome and Total Anomalous Pulmonary Venous Drainage cases hypothermic circulatory arrest at 24 °C and 26 °C was applied, respectively. Antegrade cerebral perfusion (50 mL/kg/min at 26 °C) was used for interrupted aortic arch (IAA) and aortic arch hypoplasia cases. Perfusion was achieved by anastomosing a 3.5 mm Gore-Tex graft to innominate artery; the arterial cannula was then introduced through the graft. Modified ultrafiltration was not performed in any case.

### 2.4. Statistical Analysis

Nominal variables were presented with absolute and relative frequencies (%). Continuous variables not normally distributed were described as medians with interquartile range (IQR). Normality of continuous variables was evaluated using Shapiro–Wilk test and graphical methods.

Univariate analysis was performed using chi-square test of independence or Fischer’s exact test for categorial variables, student’s *t*-test for normally distributed continuous variables, and Mann–Whitney test or Wilcoxon rank sum for not normally distributed continuous variables. Statistical significance was defined as *p* < 0.05. Assessment for interactions and collinearity was performed. For variables highly correlated, less important ones based on clinical grounds were eliminated from multivariate analysis.

Uni- and multivariate logistic regression analyses were performed to evaluate the association between risk factors and AKI development. For multivariate models variables with *p* < 0.2 from the univariate analysis were entered in a backward stepwise approach. Standard parametric or non-parametric tests as previously described were used to evaluate the association of AKI with categorical and continuous outcomes.

Kaplan–Meier survival curves were produced to visualise the overall survival of patients with and without AKI. Time was calculated from the day of surgery until the event of death. Patients discharged alive from PICU were right censored at the date of PICU discharge. Log-rank test was conducted to test for differences in the survival distributions. The level of significance was set to 0.05. To determine the effect of AKI on survival function, we performed both univariate and backward stepwise multivariate regression using Cox proportional hazard modelling. Hazard ratio (HR) was adjusted for AKI, surgical age, diagnostic category, RACHS-1 category, preoperative intubation with mechanical ventilation, and CPB in the multivariate analysis.

Data were analysed using STATA software (StataCorp. 2021. Stata Statistical Software: Release 17. College Station, TX, USA: StataCorp LLC).

## 3. Results

A total of 362 patients were included in the study. Patients’ characteristics are presented in Table 1. Patients’ detailed cardiac anatomy is presented in Table 2. AKI occurred in 70 patients (19%). Younger surgical age, lower surgical weight, longer CPB and ACC times, higher RACHS-1 category, cardiac CT scan with radiocontrast within 7 days before surgery, performance of BAS preoperatively, and preoperative mechanical ventilation were associated with higher AKI incidence postoperatively (Table 1).

Univariate logistic regression results are demonstrated in Table 3. A statistically significant difference in proportions was noted between diagnostic groups. When compared to CHD with shunt between systemic and pulmonary circulations, all other groups were associated with higher postoperative AKI incidence.

**Figure 1:**
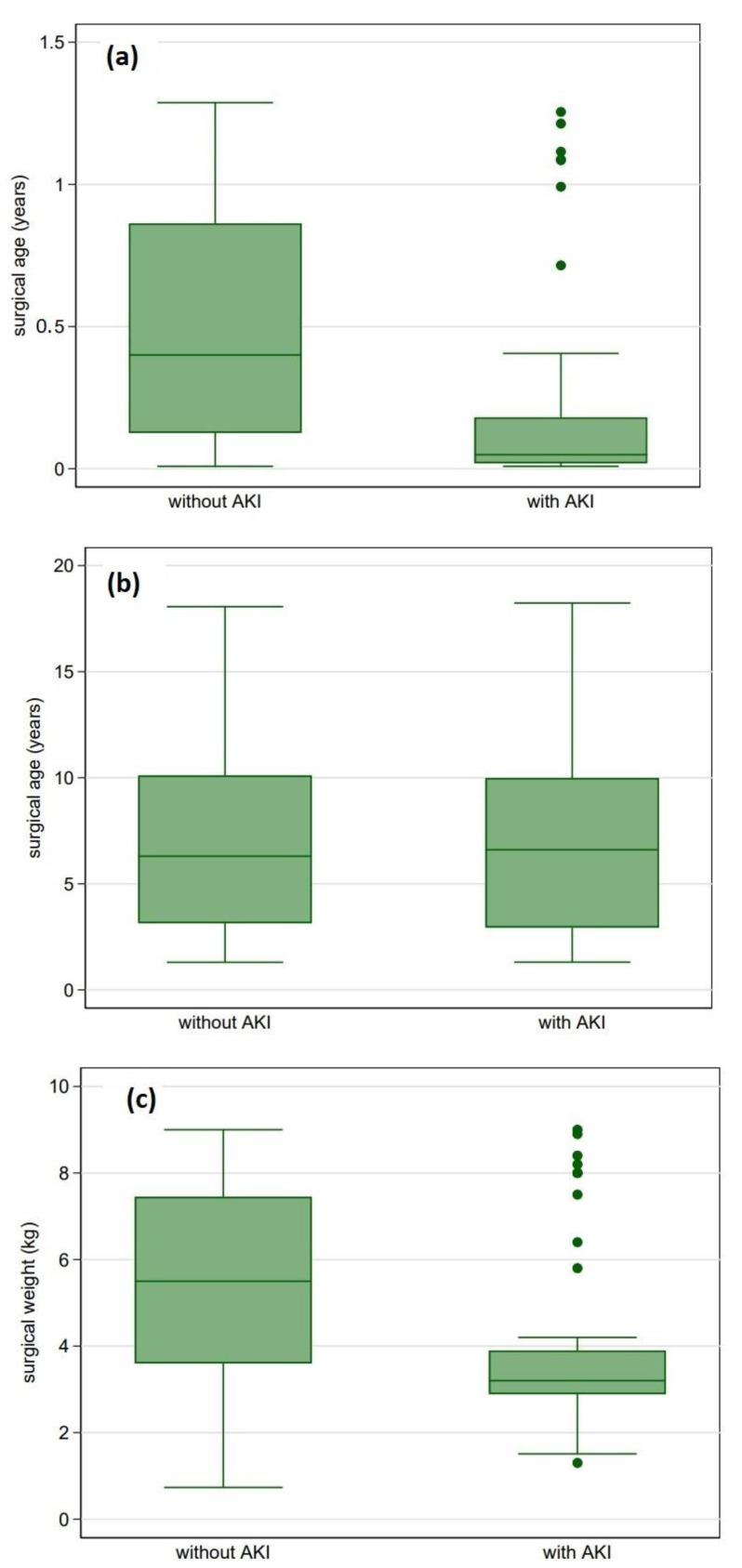

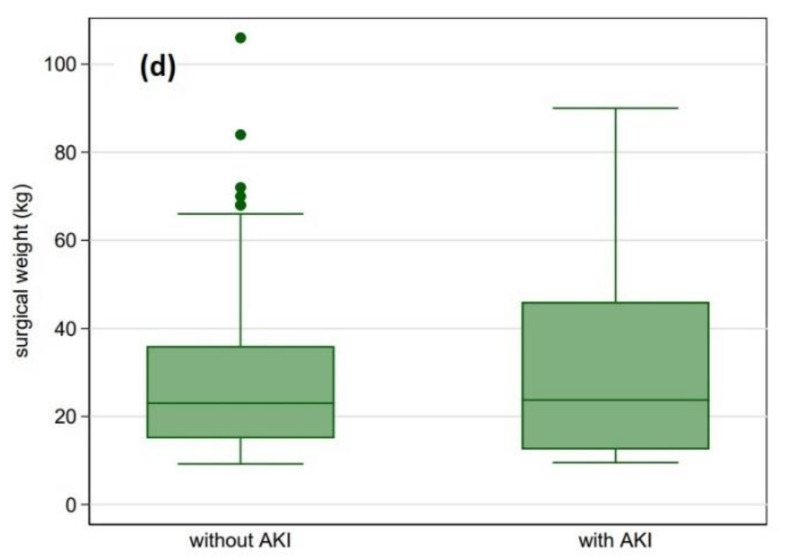
(**a**–**d**) Acute Kidney Injury association with surgical age and surgical weight. Both surgical age and weight were associated with AKI only with values below medians. (**a**) Surgical age below median vs. AKI (*p* < 0.001), (**b**) Surgical age (all values) vs. AKI (*p* = 0.7), (**c**) Surgical weight below median vs. AKI (*p* < 0.001), (**d**) Surgical weight (all values) vs. AKI (*p* = 0.74).

After adjusting for variables associated with postoperative AKI in the univariate analysis, multivariate logistic regression identified younger surgical age, longer CPB time, diagnostic category and preoperative mechanical ventilation to be associated with AKI development (Table 4, Model A). AKI association with both surgical age and weight was present only with values below medians (Figure 1a–d). When utilising both surgical age and weight as dichotomous variables, surgical age was eliminated from the multivariate model with surgical weight being associated with postoperative AKI (Table 4, Model B).

Clinical outcomes according to AKI status are shown in Table 5. Patients in the AKI group experienced longer DMV (both total and postoperative), longer PICU (total and postoperative) and hospital LOS, with all differences being significant.

**Figure 2:**
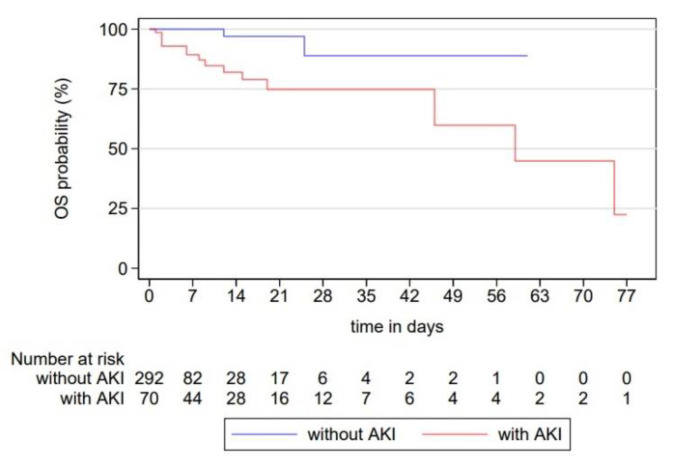
Kaplan–Meier analysis of survival in patients with and without postoperative acute kidney injury. Survival analysis showed statistically significant difference in survival between two groups (Log Rank test, *p* < 0.001), displaying a significantly higher probability of death for patients with AKI at any time point.

In total 17 (5%) patients died, 15 in the AKI and two in the non-AKI group respectively. Figure 2 displays Kaplan–Meier survival estimates for both AKI and non-AKI patients. Differences between survival distributions for the two groups were significant (log-rank test, *p* < 0.001), meaning that AKI patients had significantly higher probability of dying at any time point. A univariate Cox proportional hazard regression rendered a HR of 13.78 (95% CI 3–63.27; *p* = 0.001) for AKI group (Table 6). Adjusted multivariate HR for AKI versus non-AKI group was 11.08 (95% CI 2.45–50.01; *p* = 0.002), while for RACHS-1 group 3–6 versus RACHS-1 group 1–2 it was 4.64 (95% CI 1.03–20.9; *p* = 0.046) (Table 6).

AKI resolved in 51 out of 55 patients (93%) prior to hospital discharge. Characteristics of patients with non-resolved AKI are presented in Table 7.

## 4. Discussion

In this study, we demonstrated a 19% AKI incidence after pediatric CS for CHD, with a 21% in-hospital mortality for AKI patients. In addition to previously known risk factors, diagnostic category was identified as an independent predictor of postoperative AKI, supporting the development of CHD lesion-specific models for risk stratification. Moreover, we pointed out the negative impact of postoperative AKI on DMV and LOS, while demonstrating an increased all-cause mortality probability until PICU discharge for the AKI group, highlighting the detrimental effect of AKI on survival.

Pediatric AKI incidence after CS ranges between 11.5–62%, with reported AKI-related in-hospital mortality between 1.1–79% [12,14,18,19,20,21,22,23]. Wide incidence range could be attributed to various definitions used, differences in patient or procedural characteristics, and gradual decline in postoperative AKI risk over time [18]. A minimum SCr of >0.5 mg/dL as a prerequisite for diagnosing AKI was set, as small rises in absolute values of SCr while still <0.5 mg/dL resulting in a ≥50% percentage rise (e.g., 0.2 to 0.3 mg/dL) are of uncertain clinical importance [19].

AKI is defined as a sudden decline in renal function based on SCr levels and/or a decrease in urine output [13,24]. Limitations exist rendering both criteria insensitive for early detection of AKI, especially in neonates [24,25]. SCr is a marker of renal function rather than damage, resulting in a delay >48 h in its rise after a renal insult and a >50% drop in GFR before SCr rises [24]. Changes in renal function may be considered as a continuum of transitional stages resulting from a combination of susceptibility factors [24,26]. Several models combining multiple insults that could not otherwise cause any substantial renal injury alone have demonstrated renal medulla’s susceptibility to injury, mainly through imbalances between medullary oxygen supply and demand, membrane damage and mitochondrial dysfunction [27].

Previous studies identified surgical age and weight as risk factors for postoperative AKI [12,20,23]. In our study, AKI association with both variables was present only with values below medians, demonstrating neonatal and infantile susceptibility to renal injury. Unique features of neonatal and infant renal physiology could explain these findings, including low renal blood flow (RBF), low glomerular filtration rate (GFR), immature renal tubular function, and susceptibility to vasomotor nephropathy under stress conditions such as hypovolaemia, hypoxia, inflammation, hypothermia, and positive pressure ventilation (PPV), all of which, to some extent, are involved in pediatric surgery for CHD [24,28].

Longer CPB duration was associated with postoperative AKI, consistent with previous findings [12,14,19,20,21,29]. Multiple mechanisms have been implicated in CPB-related AKI including loss of pulsatile flow, inflammation, erythrocyte mechanical damage, haemoglobinuria, and increased production of reactive oxygen species [1,30]. Strategies to limit CPB time and further research regarding the use of vasodilator agents are needed to mitigate the detrimental effects of CPB on renal function [31].

Few studies have investigated possible associations between preoperative mechanical ventilation and postoperative AKI [23]. Preoperative mechanical ventilation could indicate a poor clinical state due to heart failure, respiratory compromise, hypoxia, and circulatory shock, conditions that could affect renal function per se. The detrimental effects of invasive ventilation on renal function should not be overlooked though. Increased intrathoracic pressures could result in decreased cardiac output and increased renal venous pressure (RVP) leading to reduced renal perfusion. PPV-related neurohormonal changes could affect renal function though redistribution of intrarenal flow from cortex to medulla, leading to reduced urine output, GFR, RBF and free water clearance. PPV is reported to increase AKI odds by a factor of three independently of diagnostic subgroups and ventilator settings [32,33,34].

Renal injury has been associated with both cyanotic and acyanotic CHD and can be present even in infancy [35]. In our study, diagnostic category was independently associated with postoperative AKI. When compared to CHD with shunt between systemic and pulmonary circulations, all other groups were associated with higher incidence of postoperative AKI potentially implicating that, depending on underlying anatomy, different pathophysiologic mechanisms affect renal function. Diagnosis per se could substantially contribute to the establishment of subclinical renal injury before AKI can be detected using available biomarkers, thus rendering children with CHD particularly susceptible to postoperative AKI. Further exposure to additional insults will gradually result in deteriorating renal function, eventually leading to AKI.

Patients with systemic-to-pulmonary shunts (i.e., left-to-right) can suffer from glomerular or tubular injury due to reduced systemic blood flow, renal hypoperfusion, and activation of sympathetic and renin–angiotensin–aldosterone systems, resulting in arterial vasoconstriction, fluid and salt retention, volume overload, heart failure, and eventually renal injury [35]. CHD with left ventricular (LV) outflow tract obstruction, such as CoA and IAA, representing 64% of our patients classified as left heart CHD, can have detrimental effects on kidney function preoperatively, mainly due to reduced RBF, decreased renal perfusion pressure (RPP), and LV dysfunction [10,35]. CHD with right ventricular (RV) outflow tract obstruction, such as tetralogy of Fallot representing 73% of our patients classified as right heart CHD, are usually characterised by RV hypertrophy and diastolic dysfunction with impaired relaxation and diastolic filling [36]. Consequently, these patients have increased RV end-diastolic and central venous pressures (CVP), with the latter representing the circulatory downstream end-pressure for systemic venous return, which must be lower than RVP for adequate RBF to the heart [37,38,39]. RV diastolic dysfunction may persist during the post-operative period contributing to post-repair increased systemic venous pressures and reduced cardiac output [36,40]. Elevated CVP increases RVP by backward transmission leading to renal congestion and has been associated with impaired renal function in a variety of clinical scenarios [38,41]. Cyanosis could potentially lead to glomerular and tubular structural damage, with a reported relationship between the risk of injury and the duration of cyanosis [35,42]. Deoxygenated arterial blood supplying the kidney can accompany many forms of CHD including mixing lesions, as well as right and left heart obstructive CHD. Renal supply for the latter ones comes through PDA by shunting deoxygenated blood from the pulmonary artery into the aorta.

Resolution of postoperative AKI prior to hospital discharge ranges between 86–100% [9,10,19,20]. Our study yielded a 93% resolution rate. All four patients with non-resolved AKI were newborns or small infants with severe CoA and postoperative residual coarctation being detected in three of them (data presented in supplementary material). We speculate residual lesions to have contributed significantly to non-resolving AKI mainly through reduced RBF and RPP.

We demonstrated prolonged DMV and LOS in the AKI group, consistent with previous reports [12,14,18,19,20,21,23]. Fluid overload due to water and salt retention, which is known to correlate with CS related AKI, can prolong the postoperative course through numerous mechanisms [1,43,44,45]. Decreased lung compliance and impaired gas exchange can result from interstitial and alveolar lung oedema. Myocardial oedema can contribute to impaired contractility and diastolic dysfunction, while gut congestion may result in feed intolerance with reduced caloric and nutrient intake [21,46]. Renal dysfunction may also affect drug elimination causing prolonged sedation, while poor wound healing can occur due to tissue oedema [46,47,48].

Although some studies report a lack of association between postoperative AKI and in-hospital mortality [10,14,20], most published data refute this finding [9,12,18,19,21]. Low rates of in-hospital deaths in aforementioned studies, rendering their power to detect significant differences in mortality inadequate, could possibly explain these reports. We demonstrated a significant difference in mortality between AKI and non-AKI patients highlighting the negative effect of AKI on survival function. The detrimental impact of AKI on survival can be explained through aforementioned associations and their consequences.

Several limitations should be considered when interpreting our findings. Firstly, its observational character precludes the establishment of any causality. Secondly, the follow-up period was limited to hospital discharge, thus no long-term outcome information is available. Thirdly, routine use of diuretics postoperatively may limit the reliability of urine output as a marker of renal injury, thus we might have missed some cases. Fourthly, as a single center study, the results need to be confirmed by larger multicenter ones. Fifthly, known risk factors such as the use of nephrotoxic drugs and fluid balance were not included in the analysis. Finally, we acknowledge that phenotypic diversity is still present within diagnostic groups due to the extremely wide spectrum of CHD. Nevertheless, we believe our study is useful for hypothesis generating considering diagnostic variation and providing an insight towards disease-specific model development.

## 5. Conclusions

CS related AKI in children with CHD had a negative impact on DMV and LOS while being associated with decreased survival to hospital discharge. Besides previously known risk factors, diagnostic category was associated with postoperative AKI. Our findings support the development of lesion-specific prognostication models for risk stratification, as significant anatomic and pathophysiologic heterogeneity in patients with CHD may hinder the ability to generalise conclusions drawn from studies conducted in patients with diverse diagnoses.

## Figures and Tables

**Table 1:** Characteristics and outcomes of the patient cohort (*n* = 362).

Characteristic	Total	Acute Kidney Injury		*p*-Value
		No	Yes	
Surgical weight (kg)	9 (4.2–23)	10.6 (6–25)	3.6 (3–8.9)	<0.001
Surgical age (years)	1.3 (0.3–6.4)	2 (0.5–7.3)	0.099 (0.02–1.41)	<0.001
CPB time (min) (*n* = 272)	89 (53–132)	79 (51–116)	146 (106–189)	<0.001
ACC time (min) (*n* = 294)	46 (23–85)	44 (23–77)	72 (32–104)	<0.001
Gender				0.53
Female	157 (43)	129 (44)	28 (40)	
Male	205 (57)	163 (56)	42 (60)	
Surgical age (≤median)	179 (49)	128 (44)	51 (73)	<0.001
Surgical weight (≤median)	184 (51)	130 (45)	54 (77)	<0.001
Previous cardiac surgery	54 (15)	44 (15)	10 (14)	0.87
Classification *^a^*				<0.001
Group 1	203 (56)	189 (65)	14 (20)	
Group 2	67 (19)	45 (15)	22 (31)	
Group 3	45 (12)	34 (12)	11 (16)	
Group 4	13 (4)	5 (2)	8 (11)	
Group 5	18 (5)	9 (3)	9 (13)	
Group 6	10 (3)	4 (1)	6 (9)	
Common Group *^b^* (4,5,6)	41 (11)	18 (6)	23 (33)	
Preoperative Mechanical Ventilation	61 (17)	30 (10)	31 (44)	<0.001
CT scan ≤ 7 days before surgery	35 (10)	18 (6)	17 (24)	<0.001
Preoperative BAS	8 (2)	4 (1)	4 (6)	0.048
RACHS-1				<0.001
1	116 (32)	106 (36)	10 (14)	
2	130 (36)	110 (38)	20 (29)	
3	89 (25)	67 (23)	22 (31)	
4	25 (7)	9 (3)	16 (23)	
5	0 (0)	0 (0)	0 (0)	
6	2 (0.6)	0 (0)	2 (3)	
RACHS-1				<0.001
1–2	246 (68)	216 (74)	30 (43)	
3–6	116 (32)	76 (26)	40 (67)	
CPB	272 (75)	223 (76)	49 (70)	0.27
ACC	294 (81)	237 (81)	57 (81)	0.96

Data are mean (%) or median (IQR). CPB = Cardiopulmonary Bypass, ACC = Aortic Cross Clamp, min. = minutes, CT = Computed Tomography, BAS = Balloon Atrial Septostomy, RACHS-1 = Risk Adjustment for Congenital Heart Surgery. *^a^* Group 1 = CHD with shunt between systemic and pulmonary circulation, Group 2 = Left heart CHD, Group 3 = Right heart CHD, Group 4 = CHD with anomalous origin of great arteries, Group 5 = Univentricular lesions, Group 6 = Miscellaneous CHD. *^b^* Common group incorporates diagnostic categories 4, 5 and 6 and was utilised in multivariate models.

**Table 2:** Patients detailed cardiac anatomy (*n* = 356).

Diagnostic Category	*n* (%)
CHD with shunt between systemic and pulmonary circulation	203 (57)
ASD	94 (26.4)
VSD	69 (19.4)
AVSD	29 (8.1)
PDA	10 (2.8)
APW	1 (0.3)
Left heart CHD	67 (18.8)
Cor triatriatum	1 (0.3)
MV stenosis	1 (0.3)
Subaortic stenosis	13 (3.7)
Aortic stenosis	3 (0.8)
Supravalvar aortic stenosis	3 (0.8)
CoA/Hypoplastic aortic arch	43 (12.1)
IAA	3 (0.8)
Right heart CHD	45 (12.6)
Ebstein’s anomaly	1 (0.3)
PS	3 (0.8)
PA/IVS	3 (0.8)
TOF	33 (9.3)
PA/VSD	5 (1.4)
CHD with anomalous origin of great arteries	13 (3.7)
TGA/IVS	7 (2)
TGA/VSD	3 (0.8)
DORV	2 (0.6)
TA	1 (0.3)
Univentricular lesions	18 (5.1)
HLHS	6 (1.7)
DORV/PS	5 (1.4)
Tricuspid Atresia	4 (1.1)
TGA/VSD/PS	1 (0.3)
DORV/PA	1 (0.3)
PA/IVS	1 (0.3)
Miscellaneous CHD	10 (2.8)
Scimitar syndrome	1 (0.3)
TAPVD	5 (1.4)
ALCAPA	4 (1.1)

CHD = Congenital Heart Disease, ASD = Atrial Septal Defect, VSD = Ventricular Septal Defect, AVSD = Atrioventricular Septal Defect, PDA = Patent Ductus Arteriosus, APW = Aortopulmonary Window, MV = Mitral Valve, CoA = Coarctation of the Aorta, IAA = Interrupted Aortic Arch, PS = Pulmonary Stenosis, PA/IVS = Pulmonary Atresia with Intact Ventricular Septum, TOF = Tetralogy of Fallot, PA/VSD = Pulmonary Atresia with Ventricular Septal Defect, TGA/IVS = Transposition of Great Arteries with Intact Ventricular Septum, TGA/VSD = Transposition of Great Arteries with Ventricular Septal Defect, DORV = Double Outlet Right Ventricle, TA = Truncus Arteriosus, HLHS = Hypoplastic Left Heart Syndrome, DORV/PS = Double Outlet Right Ventricle with Pulmonary Stenosis, TGA/VSD/PS = Transposition of Great Arteries with Ventricular Septal Defect and Pulmonary Stenosis, DORV/PA = Double Outlet Right Ventricle with Pulmonary Atresia, PA/IVS = Pulmonary Atresia with Intact Ventricular Septum, PAPVD = Partial Anomalous Pulmonary Venous Drainage, TAPVD = Total Anomalous Pulmonary Venous Drainage, ALCAPA = Anomalous Origin of the Left Coronary Artery from the Pulmonary Artery.

**Table 3:** Univariate logistic regression (*n* = 362).

Characteristic		Acute Kidney Injury		*p*-Value
		Odds Ratio (95% CI)		
Surgical weight (kg)		0.96 (0.94–0.98)		0.002
Surgical age (years)		0.87 (0.8–0.94)		0.001
CPB time (minutes)		1.01 (1.005–1.012)		<0.001
ACC time (minutes)		1.01 (1.003–1.015)		0.002
	*n* (%) with AKI		*p*	*p*
Gender				0.53
Female	28 (18)	1		
Male	42 (21)	1.19 (0.7–2.02)		
Previous cardiac surgery	10 (19)	0.94 (0.45–1.97)		0.87
Classification *^a^*				<0.001
Group 1	14 (7)	1		
Group 2	22 (33)	6.6 (3.13–13.9)	<0.001	
Group 3	11 (24)	4.37 (1.83–10.43)	0.001	
Group 4	8 (62)	21.6 (6.24–74.8)	<0.001	
Group 5	9 (50)	13.5 (4.62–39.42)	<0.001	
Group 6	6 (60)	20.25 (5.11–80.23)	<0.001	
Common Group *^b^* (4,5,6)	23 (56)	17.25 (7.58–39.23)	<0.001	
Preoperative Mechanical Ventilation	31 (51)	6.94 (3.79–12.7)		<0.001
CT scan ≤ 7 days before surgery	17 (49)	4.88 (2.36–10.08)		<0.001
Preoperative BAS	4 (50)	4.36 (1.06–17.9)		0.041
RACHS-1				<0.001
1	10 (9)	1		
2	20 (15)	1.93 (0.86–4.31)	0.11	
3	22 (25)	3.48 (1.55–7.81)	0.002	
4	16 (64)	18.84 (6.64–53.46)	<0.001	
5	0 (0)	–	–	
6	2 (100)	–	–	
RACHS-1				<0.001
1–2	30 (12)	1		
3–6	40 (35)	3.79 (2.21–6.51)		
CPB	49 (18)	0.72 (0.4–1.29)		0.27
ACC	57 (19)	1.02 (0.52–1.99)		0.96

CI = Confidence Intervals, CPB = Cardiopulmonary Bypass, ACC = Aortic Cross Clamp, min. = minutes, AKI = Acute Kidney Injury, CT = Computed Tomography, BAS = Balloon Atrial Septostomy, RACHS-1 = Risk Adjustment for Congenital Heart Surgery. *^a^* Group 1 = CHD with shunt between systemic and pulmonary circulation, Group 2 = Left heart CHD, Group 3 = Right heart CHD, Group 4 = CHD with anomalous origin of great arteries, Group 5 = Univentricular lesions, Group 6 = Miscellaneous CHD. *^b^* Common group incorporates diagnostic categories 4, 5 and 6 and was utilised in multivariate models.

**Table 4:** Multivariate logistic regression.

Characteristic	Pseudo R^2^	Acute Kidney Injury		*p*-Value
		Odds Ratio (95% CI)		
MODEL A *^a^* (*n* = 356)	0.24			
Surgical age (years)		0.91 (0.84–0.99)		0.044
CPB time (minutes)		1.01 (1.004–1.01)		<0.001
Classification *^b^*				0.003
Group 1		1		
Group 2		3.84 (1.66–8.87)	0.002	
Group 3		3.05 (1.24–7.48)	0.015	
Common Group *^c^* (4,5,6)		5.15 (1.91–13.85)	0.001	
Preoperative Mechanical Ventilation		3.38 (1.48–7.7)		0.004
MODEL B *^a^* (*n* = 356)	0.25			
Surgical Weight (kg)				0.004
>median		1		
≤median		2.97 (1.41–6.27)		
CPB time (minutes)		1.01 (1.004–1.01)		<0.001
Classification *^b^*				0.003
Group 1		1		
Group 2		3.91 (1.68–9.1)	0.002	
Group 3		2.85 (1.16–7.03)	0.023	
Common Group *^c^* (4,5,6)		5.1 (1.88–13.83)	0.001	
Preoperative Mechanical Ventilation		2.8 (1.21–6.5)		0.017

CI = Confidence Intervals, CPB = Cardiopulmonary Bypass. *^a^* Model A utilises surgical age and weight as continuous variables, while Model B as dichotomous (≤median and >median). *^b^* Group 1 = CHD with shunt between systemic and pulmonary circulation, Group 2 = Left heart CHD, Group 3 = Right heart CHD, Group 4 = CHD with anomalous origin of great arteries, Group 5 = Univentricular lesions, Group 6 = Miscellaneous CHD. *^c^* Common group incorporates diagnostic categories 4, 5 and 6 and was utilized in multivariate models.

**Table 5:** Clinical outcomes according to AKI.

Characteristic	Total	Acute Kidney Injury		*p*-Value
		No	Yes	
		Median (IQR)		
DMV, post operative(days)	2 (1–4)	2 (1–3)	6 (2–12)	<0.001
DMV, total (days)	2 (1–5)	2 (1–3)	8 (3–18)	<0.001
ICU length of stay, postoperative (days)	4 (2–8)	3 (2–7)	9.5 (5–20)	<0.001
ICU length of stay, total (days)	4 (2–11)	3 (2–8)	13.5 (6–26)	<0.001
Hospital length of stay (days)	12 (9–22)	10 (8–19)	23.5 (14–44)	<0.001

AKI = Acute Kidney Injury, IQR = Interquartile Range, DMV = Duration of Mechanical Ventilation, ICU = Intensive Care Unit.

**Table 6:** Univariate and multivariate Cox regression according to postoperative Acute Kidney Injury development.

	Univariate Cox Regression (*n* = 362)		Multivariate Cox Regression (*n* = 356)	
Characteristic	Hazard Ratio (95% CI)	*p*-Value	Hazard Ratio (95% CI)	*p*-Value
Acute Kidney Injury	13.77 (3–63.27)	0.001	11.08 (2.45–50.01)	0.002
RACHS-1		0.019		
1–2	1		1	
3–6	6.33 (1.36–29.42)		4.64 (1.04–20.9)	0.046
Classification *^a^*		0.019		
Group 1	1			
Group 2	5.8 (0.6–56.55)			
Group 3	11.85 (1.3–107.66)			
Common Group *^b^* (4,5,6)	12.3 (1.43–105.46)			
Preoperative Mechanical Ventilation		0.053		
No	1			
Yes	2.95 (0.99–8.82)			
CPB time (min)	1.01 (1.003–1.02)	0.002		
Weight (kg)	0.91 (0.79–1.04)	0.16		

CI = Confidence Intervals, RACHS-1 = Risk Adjustment for Congenital Heart Surgery. *^a^* Group 1 = CHD with shunt between systemic and pulmonary circulation, Group 2 = Left heart CHD, Group 3 = Right heart CHD, Group 4 = CHD with anomalous origin of great arteries, Group 5 = Univentricular lesions, Group 6 = Miscellaneous CHD. *^b^* Common group incorporates diagnostic categories 4, 5 and 6 and was utilised in multivariate models.

**Table 7:** Characteristics of patients with non-resolved AKI.

Patient No	SW	SA	Diagnosis	CPB	ACC	Classification	RACHS-1	Preop IPPV	Cardiac CT Scan	DMV Postop	DMV Total	ICU Postop	ICU Total	Hospital LOS
1 *^a^*	2.5	0.08	CoA	N/A	24	2	2	No	No	4	4	7	7	28
2 *^b^*	4.2	0.18	CoA	N/A	16	2	2	No	No	3	3	10	10	41
3	2.8	0.01	CoA	N/A	16	2	2	Yes	Yes	3	4	5	9	12
4 *^c^*	1.3	0.01	CoA	N/A	20	2	2	Yes	No	5	6	6	8	8

AKI = Acute Kidney Injury, SW = Surgical Weight, SA = Surgical Age, CPB = Cardiopulmonary Bypass, ACC = Aortic Cross Clamp, RACHS-1 = Risk Adjustment for Congenital Heart Surgery, Preop = Preoperative, IPPV = Invasive Positive Pressure Ventilation, CT = Computed Tomography, DMV = Duration of Mechanical Ventilation, Postop = Postoperative, ICU = Intensive Care Unit, LOS = Length of Stay, CoA = Coarctation of the Aorta. *^a^* IUGR; presentation at 30 days old. *^b^* presentation at 67 days old. *^c^* prematurity 34^+1^/40 weeks; Very Low Birth Weight; twin. Underwent subclavian flap repair after failed end-to-end anastomosis. Discharged to another hospital for further neonatal care. AKI was not resolved at the time of hospital discharge.

## Data Availability

The datasets generated and analyzed from this study are available from the corresponding author upon reasonable request.
